# N-Acetylglucosamine Sensing and Metabolic Engineering for Attenuating Human and Plant Pathogens

**DOI:** 10.3390/bioengineering9020064

**Published:** 2022-02-05

**Authors:** Sekhu Ansari, Vinay Kumar, Dharmendra Nath Bhatt, Mohammad Irfan, Asis Datta

**Affiliations:** 1 Division of Pathology, Cincinnati Children’s Hospital Medical Center, Cincinnati, OH 45229, USA; shekhuansar@gmail.com; 2 Department of Physiology and Cell Biology, Department of Internal Medicine, The Ohio State University Wexner Medical Center, Columbus, OH 43210, USA; vinayktyagi07@gmail.com; 3 National Institute of Plant Genome Research, Aruna Asaf Ali Road, New JNU Campus, New Delhi 110067, India; mashbh101@gmail.com; 4 Plant Biology Section, School of Integrative Plant Sciences, Cornell University, Ithaca, New York, NY 14453, USA

**Keywords:** N-Acetylglucosamine, pathogens, virulence, colonization, NAG1, DAC1, HXK1, NGT1, chitin, plant immunity

## Abstract

During evolution, both human and plant pathogens have evolved to utilize a diverse range of carbon sources. N-acetylglucosamine (GlcNAc), an amino sugar, is one of the major carbon sources utilized by several human and phytopathogens. GlcNAc regulates the expression of many virulence genes of pathogens. In fact, GlcNAc catabolism is also involved in the regulation of virulence and pathogenesis of various human pathogens, including *Candida albicans*, *Vibrio cholerae*, *Leishmania donovani*, *Mycobacterium*, and phytopathogens such as *Magnaporthe oryzae*. Moreover, GlcNAc is also a well-known structural component of many bacterial and fungal pathogen cell walls, suggesting its possible role in cell signaling. Over the last few decades, many studies have been performed to study GlcNAc sensing, signaling, and metabolism to better understand the GlcNAc roles in pathogenesis in order to identify new drug targets. In this review, we provide recent insights into GlcNAc-mediated cell signaling and pathogenesis. Further, we describe how the GlcNAc metabolic pathway can be targeted to reduce the pathogens’ virulence in order to control the disease prevalence and crop productivity.

## 1. Introduction

Pathogens have developed numerous strategies for successful host colonization, which depends upon their ability to cope with a poor nutritional environment for the pathogens and stressful conditions inside the host. The pathogenesis mechanism involves multifarious parallel metabolic pathways, complex gene regulatory network systems, and stress adaptive mechanisms to survive inside the host [1]. During infection, many human pathogens target phagocytic cells, especially macrophages [2]. In the defense mechanism against pathogens, macrophages apply several stress mechanisms like oxidative and nitrosative stress to kill the pathogens [3]. Pathogens evade this host defense mechanism to reach the parasitophorous vacuole. To restrict the growth of phagocytosed pathogens, phagocytic cells maintain a low level of glucose inside their parasitophorous vacuole (PV) [2]. PV has a nutritionally poor and stressful environment. One of the adaptative strategies that evolved in pathogens is their capability to utilize a diverse range of carbon sources for their survival under hostile environments [1]. Under these circumstances, the parasites living inside the PV depend heavily on the amino sugars present inside the PV. Amino sugars (N-acetylglucosamine and Glucosamine) are a major class of non-conventional sugars consumed by pathogens in the absence of conventional carbon sources. Therefore, the utilization of N-acetylglucosamine (GlcNAc) as a carbon source under hostile conditions is an important adaptive mechanism found in many human pathogens [4,5,6,7]. Most of the cells could not synthesize non-phosphorylated GlcNAc. Therefore, GlcNAc is synthesized de novo either, in the form of GlcNAc-6-P or GlcNAc-1-P, inside the cell. Hence, the free form of GlcNAc is never expected inside the cell unless it is taken up from the outside source. Prokaryotes synthesize GlcNAc-1-P, while eukaryotic cells synthesize GlcNAc-6-P [8]. Accordingly, depending upon the phosphorylation or non-phosphorylation of GlcNAc, cells distinguish between the endogenous and exogenous GlcNAc.

In plants, pathogenic fungi, which severely affect crop production, change the host metabolism upon fungal invasion [9,10,11,12,13,14]. Fungal pathogens either kill the host cells to get nutrients by consuming the dead tissues in a necrotrophic or establish a long-term feeding association with the host instead of killing the host cells in a biotrophic interaction fashion [15,16,17,18]. During pathogenesis, the host cell death or hypersensitive response is caused by reactive oxygen species (ROS) and used by the host as a defense tool against biotrophic pathogens. The hypersensitive response-mediated cell death or resistance against biotrophs can result in increased susceptibility against necrotrophic pathogens and vice versa [19,20]. Thus, hosts employ different defense strategies depending on the nature of pathogens [21,22,23]. The fungal cell wall contains chitin, a GlcNAc polymer, and is synthesized by chitin synthases using UDP-GlcNAc, generated from different sugars. Chitin is broken down into its monomers during fungal cell wall remodeling [8]. Therefore, GlcNAc is an important component during chitin metabolism, cell wall remodeling, and host colonization by fungal pathogens. 

In this review, we provide recent insights into GlcNAc sensing and signaling mechanisms in different human and plant pathogens. We discuss how the GlcNAc metabolic pathway has been targeted to reduce the virulence of these severe pathogens using different molecular genetics, biotechnology, and genome engineering tools. Moreover, the prospects of GlcNAc and its metabolic pathway genes as tools for drug discovery and plant immunity have also been discussed. 

## 2. GlcNAc: A Ubiquitous Amino Sugar and Signaling Molecule

**Figure 1:**
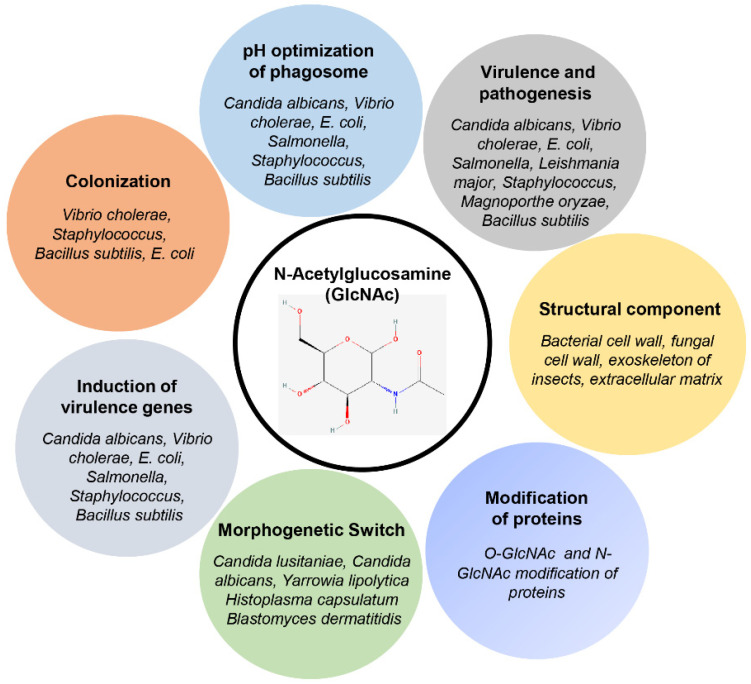
Diverse roles of GlcNAc. The GlcNAc plays a key role in pathogenesis and provides a survival advantage to the pathogens in the host. The chemical structure of GlcNAc (PubChem CID 439174) retrieved on 19 January 2022 from https://pubchem.ncbi.nlm.nih.gov/compound/N-Acetyl-D-Glucosamine.

**Figure 2:**
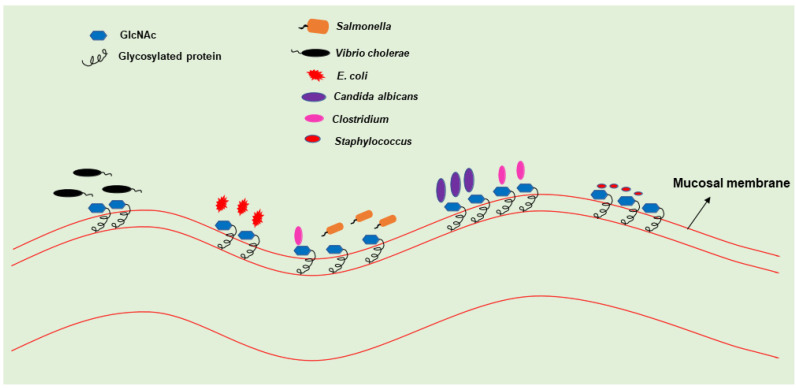
Pathogen infection at mucosal membrane: Mucous membrane is rich in glycosylated proteins. Several pathogens such as *Candida albicans*, *E. coli*, *Salmonella* spp., *Vibrio cholerae*, etc., exploit GlcNAc released from glycoproteins at the mucosal membrane.

The ubiquitous presence of the GlcNAc makes it among the most abundant sugars on Earth. GlcNAc is present in almost all life forms present on Earth, such as archaebacteria, mycoplasma, bacteria, fungi, plants, and animals [8]. GlcNAc has a diverse role in different biological processes, as shown in Figure 1. GlcNAc is particularly known for its structural role in distinct cell types across living organisms. In bacteria, GlcNAc provides structural support by forming the backbone of the cell wall in the form of peptidoglycan [8,24]. In fungi, GlcNAc provides structural support in the form of chitin [8]. In multicellular organisms, the intercellular space is packed with an organized meshwork of extracellular matrix. It provides structural and biochemical support to the cells. The extracellular matrix contains a wide range of sugar polymers like glycosaminoglycans, heparin sulfate, and keratin sulfate [25]; most have GlcNAc as a monomer. Among these macromolecules, glycosaminoglycans form a major part of the extracellular matrix in humans. Besides the extracellular matrix, GlcNAc is also abundantly present at mucosal sites like the gastrointestinal tract and vaginal and oral thrush [26]. These mucosal sites are often the site of entry inside the body for several pathogens. Several pathogens, including *C. albicans*, colonize the mucosal membranes of the GI tract, oral cavity, and vaginal thrush (Figure 2; Table 1) [27,28]. GlcNAc is also present in plants, albeit in trace amounts. However, it is mainly found in the form of glycosylated proteins [29]. In plants, many studies have shown that different free N-glycans in the common core (Man3Glc NAc2) regulate various biological processes [30,31,32,33,34]. Plant membrane sphingolipids, e.g., glucosamine inositol phosphorylceramide, also contain GlcNAc and are involved in cell-cell adhesion [35].

During infection, pathogens secrete a huge amount of hyaluronidases to release the GlcNAc present in the mucosal membrane and ECM [7]. This GlcNAc is used as a carbon source by pathogens during the initial penetration of the human body. Hyaluronidase secretion also stimulates the recruitment of the phagocytic cells to the site of infection. Pathogens are then taken up by macrophages. Inside the macrophages, these pathogens end up being inside the phagolysosomal compartment. These compartments are acidic and rich in hydrolytic enzymes [50]. These phagolysosomal compartments regularly receive waste macromolecules of the cells like glycoproteins, proteoglycans, and glycolipids [1,2,51]. These macromolecules are then degraded by the hydrolases to release the GlcNAc. Since macrophages regularly maintain low glucose levels, pathogens depend heavily upon the GlcNAc released from macromolecules. Therefore, the ubiquitous presence of GlcNAc from the cell surface to the extracellular matrix to the inside of the cell makes it one of the most important sugars for the pathogen. Moreover, numerous proteins in the cytoplasm and nucleus are also regulated by GlcNAc, probably at serine and threonine residues (O-GlcNAc) by the O-GlcNAc transferase [52]. GlcNAc is also reported as an inducer of *Candida albicans* morphological plasticity (yeast to hyphae transition), a pathogenic trait as the hyphal form has significant roles in the infection process [53]. To understand this, various proteomics approaches have been employed, and many GlcNAc-regulated protein and phosphoproteins involved in morphological transition were identified [54,55].

There is growing evidence that GlcNAc acts as an intracellular signaling molecule in signaling pathways that impact the virulence and survival of the pathogens in hosts such as *Candida albicans* [24,56]. The first report of GlcNAc as a signaling molecule was reported in the human fungal pathogen *C. albicans*. GlcNAc induces the switching of the budding yeast form to the filamentous hyphal form in *C. albicans* [53]. This switching in morphology is c-AMP-mediated. Later, this role of GlcNAc-induced morphogenesis into the filamentous form was discovered in several other fungal pathogens. Hyphal forms of fungus show invasive growth in hosts. GlcNAc also induces several genes (aspartyl proteases, phospholipases, adhesins, etc.) involved in the virulence and biofilm formation in *C. albicans* [8]. Besides providing the energy, GlcNAc catabolism plays a vital role in raising the pH of the acidic phagolysosome, thereby providing the ambient pH for its survival [57]. GlcNAc also induces the galactose metabolic pathway in *C. albicans*, which is very unusual as both catabolic pathways do not overlap [58].

In bacterial pathogens, GlcNAc regulates several virulence factors. In *E. coli*, GlcNAc downregulates the expression of the fimbriae and curli fibers, which are important virulence determinant factors [8]. GlcNAc also regulates the production of secondary metabolites in bacteria. Production of antibiotics by the soil bacteria and phenazine (antimicrobial compound) by *P. aeruginosa* is induced by the GlcNAc-mediated signaling [8]. In pathogenic bacteria, *Listeria monocytogenes* flagellar motility is modulated by the O-GlcNAcylation of the flagellar protein [59]. In the mammalian system, GlcNAc is a major constituent of the ECM. GlcNAc is released during the remodeling of ECM or the parasitic infection through hyaluronidases; a significant amount of GlcNAc is released, which acts as a signaling molecule for both mammalian cells and parasites [7]. Several mammalian cell surface proteins regulate the cell signaling pathways by altering the pattern of N-glycosylation. GlcNAc has also been found to influence the mammalian immune cells. GlcNAc inhibits the T-cell response. The Th1 and Th17 cell-response against the fungal defense is inhibited by GlcNAc [60]. Not much is known about GlcNAc inhibition of the T-cell response. 

Depending upon the metabolic requirements of the cells, GlcNAc can either enter the catabolic pathway for energy generation, or it can enter the anabolic pathway for the biosynthesis of other cellular metabolites. Amino sugars are required for the biosynthesis of a wide range of surface glycoconjugates and N-glycosyl modification of proteins. This role of amino sugars is well established from lower prokaryotes to higher eukaryotes [8]. GlcNAc can be converted to UDP-GlcNAc, and this UDP-GlcNAc is used by the cell for the biosynthesis of several glycoconjugates like glycosylphosphatidylinositol (GPI), dolichol-linked oligosaccharides (DLO), gp63, PSA2, LPG. UDP-GlcNAc is also essential for the glycosylation of proteins. The biosynthesis of UDP-GlcNAc is also crucial for the survival of several pathogens. UDP-GlcNAc biosynthesis is critical for the survival of Trypomastigote—the bloodstream form of *T. brucei* [61]. UDP-GlcNAc synthesis is important for the intracellular survival of *Mycobacteria tuberculosis* [62]. In many bacterial and fungal pathogens, UDP-GlcNAc acts as a GlcNAc donor for the cell wall synthesis and glycosylation of proteins [8]. 

## 3. Universality of GlcNAc Catabolic Pathway and Genes in Human and Plant Pathogens

**Figure 3:**
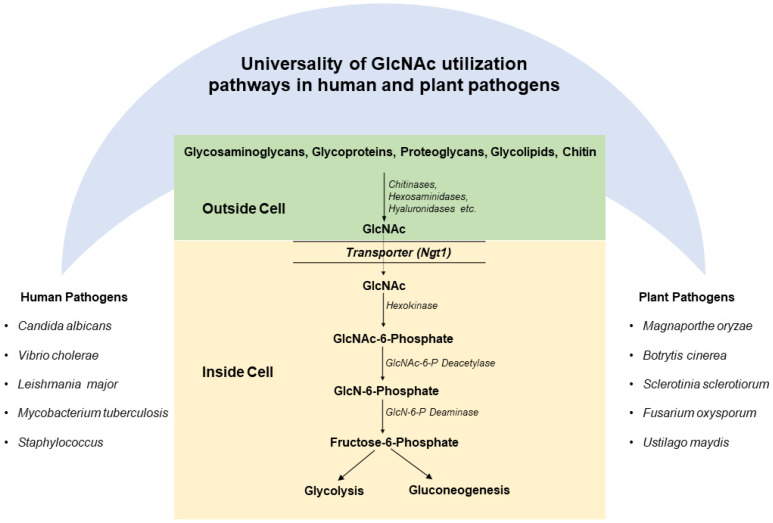
GlcNAc catabolic pathway: glycosaminoglycans, glycoproteins, proteoglycans, glycolipids, and chitin are the major sources of GlcNAc. Enzymes such as chitinases, hexosaminidases, hyaluronidases, etc., release free-GlcNAc from these macromolecules, which are taken up by the pathogens. The GlcNAc catabolic pathway involves three enzymes: hexokinase, GlcNAc-6-P deacetylase, and GlcNAc-6-P deaminase, which act sequentially to convert GlcNAc into fructose-6-phosphate.

Various studies demonstrate that the GlcNAc catabolic pathway is evolutionary conserved in human and plant pathogens (Figure 3). The genes of the GlcNAc catabolic pathway have been identified in human bacterial pathogens such as *Mycobacterium*, *Vibrio cholerae*, yeast ascomycetes, filamentous ascomycetes, basidiomycetes and zygomycetes fungi, and protozoan parasites [1,4,52,63]. Similarly, the genes involved in GlcNAc utilization are identified in the genomes of several phytopathogenic fungi, including *M. grisea*, *Gibberella zeae, Ustilago maydis*, *Sclerotinia sclerotiorum,*, *Botryotinia fuckeliana*, *Pyrenophora tritici-repentis*, *Cochliobolus heterostrophus*, *Mycosphaerella fijiensis*, *Nectria haematococca*, and *Aspergillus niger* [64,65]. This unique nature of the GlcNAc catabolic pathway suggests an important role of the gene cluster in pathogenesis. In pathogens, including *V. cholera*, *C. albicans*, and *M. oryzae*, the GlcNAc catabolic pathway genes are present as a cluster in an operon [52]. In *C. albicans*, GlcNAc catabolic pathway genes are clustered together on chromosome 6 [52]. However, in protozoan parasites, like *L. donovani*, these genes are present on separate chromosomes [1,2].

Free GlcNAc is released by the glycosidases, such as is taken up the bacterial cells through their phosphotransferase system (PTS), while eukaryotes have specific transporters for the GlcNAc [8,66]. For pathogens such as *C. albicans*, *M. oryzae* has *Ngt1* for GlcNAc import (Figure 3). Inside the eukaryotic cells, three enzymes, namely: hexokinase (HXK), N-acetyl glucosamine-6-phosphate deacetylase (DAC), and glucosamine-6-phosphate deaminase (NAG), function in a sequential manner in the catabolism of GlcNAc (Figure 3) [8,52,65]. These three enzymes are highly conserved in pathogens. GlcNAc is phosphorylated by HXK to form N-acetylglucosamine-6-phosphate (GlcNAc-6-P) [7,8,52]. GlcNAc-6-P is then deacetylated by DAC to form the glucosamine-6-phosphate (GlcN-6-P) [52,65]. Further, GlcN-6-P is deaminated and isomerized by NAG to form fructose 6-phosphate [52,65]. Fructose-6-phosphate is a common metabolic intermediate having multiple fates, which can be used for generating the energy requirement of the cell [8,52,65]. In most pathogens, GlcNAc catabolism closely follows the glycolytic pathway. Therefore, the GlcNAc catabolism takes place in the cytoplasm of the cell. However, in pathogens, like *Leishmania*, *Trypanosoma* glycolytic enzymes are localized in glycosomes. Thus, in these pathogens, GlcNAc catabolism takes place in glycosomes [1,2].

## 4. Engineering GlcNAc Catabolic Pathway for Reducing the Virulence of Human Pathogens

Genetic engineering of human pathogens has contributed significantly to our understanding of human health and diseases. Since the emergence of knockout strategies, they have been the most commonly and widely used method for establishing an understanding of human pathogens. The emergence of more efficient knockout generation technologies, like CRISPR, has contributed considerably to the genetic engineering of pathogens [67]. *C. albicans* was among the first human pathogens in which the GlcNAc metabolic pathway was thoroughly studied, and Bhattacharya et al. [68] first reported that the presence of GlcNAc is essential for the induction of GlcNAc kinase. Further, several other GlcNAc-regulated metabolic enzymes, such as permease, GlcNAc-6-P deacetylase, and GlcNAc-6-P deaminase in spheroplasts of *C. albicans*, were studied [69]. Moreover, Kumar et al. [70] reported that in *C. albicans*, GlcNAc catabolic genes (*NAG1*, *DAC1*, and *HXK1*) exist as a cluster and are under transcriptional activation in response to a single inducer GlcNAc [70]. Interestingly, this *NAG* cluster contains *NAG1* and *DAC1* genes in opposite orientations, indicating the possibility of a bidirectional promoter [70]. The role of the GlcNAc catabolic pathway in virulence and pathogenesis was first established in *C. albicans* through the generation of several knockouts [28]. Disruption of the *NAG* operon of *C. albicans* by deletion of *DAC1*, *NAG1*, or *HXK1*, results in the generation of strain, which has attenuated virulence and pathogenesis [27,28]. Emerging data indicate that GlcNAc is utilized and sensed by a broad range of pathogens. The ubiquitous presence of GlcNAc and its role in inducing the virulence factors in many fungal and bacterial pathogens suggest the much-needed attention this pathway requires in several important human and plant pathogens. 

After the establishment of the fact that the GlcNAc catabolic pathway is essential for the virulence and pathogenesis of *C. albicans*, this pathway was explored in other human pathogens. In *Vibrio cholerae*, two copies of DAC and GlcNAc kinase were identified, and an N-acetylglucosamine-specific repressor (*NagC*) performs dual functions by regulating classical GlcNAc catabolic pathway genes negatively and second copies of these genes positively. The null mutants of GlcNAc catabolic pathway genes showed attenuated virulence and pathogenesis during intestinal colonization. GlcNAc catabolic engineering approach was further exploited in a protozoan parasite *Leishmania donovani*. The Δ*nagd* mutant of *Leishmania donovani* showed impaired GlcNAc catabolism and was reported as indispensable for the viability of *L. donovani* in media containing GlcNAc as the sole carbon source [1]. Moreover, Δ*nagd* mutant exhibited attenuated virulence and reduced proliferation rate in THP-1 cells as compared to wild type [1]. Recently, null mutants of the GlcNAc catabolic pathway were also created in several other human pathogens such as *S. mutans*, *Mycobacteria*, *Salmonella*, etc., which also exhibited attenuated virulence and pathogenesis [6,48,49,71]. 

## 5. GlcNAc Metabolic Engineering for Reducing the Virulence of Plant Pathogens

**Figure 4:**
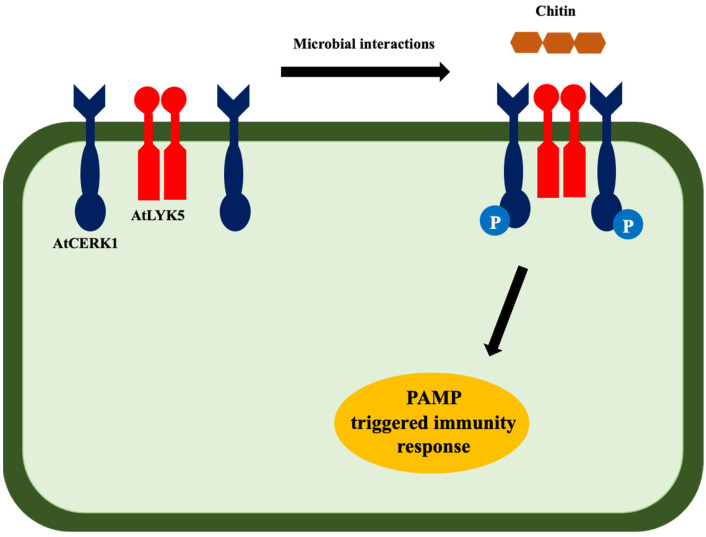
Chitin-triggered immunity in plants. Microbe-derived chitin activates the formation of tetramer [AtCERK1(AtLYK5)2 AtCERK1] that phosphorylates the AtCERK1 at the same time. This phosphorylation activates the PAMP-triggered immunity in plants [78].

Similar to vertebrates and other animals, plants also interact with pathogens; however, plants do not have specialized immune cells. To combat this, plants have evolved intracellular immune receptors to recognize the presence of pathogen-associated molecular patterns (PAMPs) [72,73,74]. Bacterial and fungal cell-derived N-acetylglucosamine biopolymer is among the PAMP molecules that activate pattern-triggered immunity (PTI) in plants (Figure 4) [75]. Chitin, a polymer of GlcNAc and structural component of the fungal cell wall, triggers two major lysin motif receptor-like kinases, AtLYK5 and AtCERK1, in *Arabidopsis* [76]. AtLYK5 has a higher binding affinity to chitin than AtCERK1. Research studies show that chitin treatment activates the formation of tetramer complex, which further activates the chitin-triggered immunity in plants [77,78]. 

During the establishment of disease in plants, pathogen (microbe) and host (plant) interactions are tightly regulated at the molecular level. This pathological interaction not only hampers plant growth but also obstructs reproduction. It is important to know that to synthesize cell surface structures in bacteria, GlcNAc plays an important role. On entering the glycolytic pathway, GlcNAc could supply both carbon and energy as it can be converted into fructose-6-phosphate [50,51]. Kashulin et al. performed a study on tissue cultured potato cells with a fluorescent dye 2′,7′-dichlorofluorescein diacetate to understand the outcomes of polyunsaturated fatty acids together with GlcNAc and reported rapid generation of H_2_O_2_ in the cells [79]. This rapid (within 2–10 min) spur of free radicals may be associated with a plant safeguarding approach domineering a plant-pathogen collaboration. To combat such pathogenic assault, many plants have developed defense proteins, which contain ‘hevein domain’ or ‘chitin-binding’ motif, and are skilled in reversible binding to polysaccharide chitin, a long-chain polymer of GlcNAc, present in the cell wall of fungi [80]. Asensio et al. employed various biochemical and spectroscopic approaches to understand the interactions of GlcNAc oligomers with hevein and ultimately established a structural foundation for detection of chitin by plant defense proteins [81]. 

Occurrence and/or omission of similar genes involved in plant resistance and pathogen avirulence governs plant-pathogen interactions. Kooman-Gersmann et al. applied AVR9 mutant peptides to establish the association among elicitor activity of AVR9 peptides and their affinity to bind tomato membranes [82]. Interestingly, they observed that N-glycosylation plays an important role, as AVR9 peptides with N-glycosylation exhibited a lesser affinity to the binding site than the non-glycosylated AVR9 peptides, while their necrosis stimulating endeavor was barely altered [82,83]. Additionally, tomato pathogen *Cladosporium fulvum* manifests *AVR4* to protect fungi against plant chitinases [84,85,86]. Further, the importance of class V chitin synthase in safeguarding vascular wilt pathogen *Fusarium oxysporum* against plant defense during host infection was also studied [87,88]. Jaroszuk-Ściseł et al. studied *Fusarium* isolates and their enzymatic complexes in the hydrolysis of the plant cell wall and/or fungal cell wall with distinctive assertiveness to *Secale cereale* and established that a plant growth-promoting rhizosphere isolate was effective in emancipating GlcNAc and reducing sugars from the cell wall of fungi [89,90]. 

Diverse soil microbes comprising symbionts and pathogens interact with plant roots, which advanced receptors to sense microbe-derived molecules. This mechanism helps plants to either allow the beneficial microbe to institute symbiosis or subsidize to decline a pathogen to prevent plant disease. Among the receptors, plant lysin motif proteins, which regulate the sensitivity of microbial-derived GlcNAc compounds, play a functional role in deciding symbiosis or preventing plant disease. Rey et al. studied LysM-receptor-like kinase mutants (*nfp* and *lyk3*) of *Medicago truncatula* and examined them by subsequent inoculation with a root oomycete, *Aphanomyces euteiches* [91]. They found that *nfp* (Nod Factor Perception) mutants show greater susceptibility to *A. euteiches*, while *NFP* overexpression enhances resistance to *A. euteiches*; however, they did not find any change in *lyk3* (LysM domain receptor-like kinase 3) [91]. Further, the molecular basis for *LysM* modules in recognizing polysaccharides containing GlcNAc residues from *AtlA*, an autolysin of bacterial pathogen *Enterococcus faecalis*, was explored, and it was discovered that the LysM module distinguishes that the GlcNAc-X-GlcNAc motif exists in polysaccharides throughout all kingdoms [92]. Moreover, lysin motif receptors are also reported to be involved in the perception of GlcNAc-based saccharides released by pathogenic fungi [93]. Nars et al. reported the structural characterization and biological activity of atypical chitosaccharides from *A. euteiches* on the host *Medicago truncatula* and found that expression of defense marker genes is induced by glucan-chitosaccharide fractions of *A. euteiches* in *Medicago* [94]. Moreover, enhanced resistance from fungal pathogens by over-expressing chitinase genes have been successfully engineered in crop plants such as *Solanum lycopersicum*, *Solanum tuberosum*, *Oryza sativa*, *Zea mays*, groundnut, *Brassica*, finger millet, cotton, lychee, banana, *Vitis vinifera*, and wheat [95,96,97,98]. Furthermore, chitin-binding lectins (CBLs) are known for their role in immune defense against chitin encompassing pathogens by displaying antifungal properties as recombinant CBL in the 293F cell culture supernatant could inhibit the growth of *Rhizoctonia solani* and *Colletotrichum gloeosporioide* [99]. Fungi are known for their capability to secrete extracellular degrader enzymes with hydrolytic functions. Barreto et al. suggest that GlcNAc has an important role in the secretion of extracellular chitinase in *Metarhizium anisopliae* [100,101]. 

During host-pathogen interactions, the successful establishment of pathogens is mostly dependent on accessibility and proficient consumption of host-derived nutrients by pathogens. To better understand this, Kumar et al. characterized the GlcNAc catabolic pathway genes such as GlcNAc transporter (*MoNgt1*), hexokinase, GlcNAc-6-phosphate deacetylase (*MoDac*), and GlcN-6-phosphate deaminase (*MoDeam*) during phytopathogen *M. oryzae* communication with host rice plant [65]. They proposed that GlcNAc supports fungus by the antioxidant defense to astounded oxidative stress inside its host, while in the impaired catabolic pathway, GlcNAc becomes toxic to the *M. oryzae* [65]. In line with this work, Bhatt et al. acknowledged a conserved transcriptional regulator, *Ndt80/PhoG*, in GlcNAc catabolic gene cluster in various fungi, including *M. oryzae* [64]. Interestingly, it was reported that *MoNdt80* is indispensable for GlcNAc utilization and successful colonization and pathogenesis of *M. oryzae* in its host [64]. Other studies also support the role of GlcNAc metabolism (in planta and in vitro) in *Xanthomonas campestris*, a causal agent of black rot disease on Brassica [102,103,104]. To decode the molecular mechanism of *Fusarium oxysporum* and its host during infection, López-Fernández et al. discovered the presence of seven putative N-glycosyl transferase encoding genes named *gnt* [105]. The Δ*gnt2* mutant exhibited a decline in virulence on both plant and animal hosts, and the authors have concluded that N-acetylglucosaminyl transferases are not only essential factors for cell wall structure but also stimulus interactions of *F. oxysporum* [105,106]. 

Oligosaccharides with mixed linkages, β-1,3/1,4-glucan (β-1,3/1,4-MLG), are plentifully found in bacteria, oomycetes, symbionts, pathogenic or non-pathogenic fungi, and monocot plants. Barghahn et al. examined mutants of *Arabidopsis* innate-immunity signaling as well as more than 100 *Arabidopsis* ecotypes and hypothesized that β-1,3/1,4-MLG oligosaccharides contain the two-fold capability to perform as immune active microbe-associated molecular patterns (MAMPs) and danger-associated molecular patterns (DAMPs) in both monocot and dicot plants [107]. Additionally, Rebaque et al. suggested that MLGs work as a cluster of carbohydrate-established molecular patterns and are noticed by plants to initiation their immune reactions and disease endurance [108,109]. Furthermore, three of the eight members of the LysM pattern recognition receptor (*PRR)* family, i.e., *CERK1*, *LysM domain receptor-like kinase-4* (*LYK4*), and *LysM domain receptor-like kinase-5 (LYK5)* are involved in (GlcNAc)4–8 perception by *Arabidopsis*. *Chitin Elicitor Receptor Kinase 1 (CERK1)* identifies bacterial peptidoglycan MAMPs and chito-oligosaccharides from fungi to work as an immune co-receptor for linear 1,3-β-d-glucans [110].

## 6. Conclusions and Future Prospects

The emerging role of the GlcNAc catabolic pathway in different human and plant pathogens establishes the importance of this pathway in pathogenic diseases. Due to the continuous emergence of drug resistance among the parasites, there is a continuous search for newer drug targets. Therefore, studies on GlcNAc metabolic pathway in various human pathogens have provided the basis for new drug targets to manage microbial diseases. Recently characterized GlcNAc pathway in phytopathogen *M. oryzae* suggests the universality of this pathway in human and plant pathogens. Thus, it is clear that GlcNAc is universally required for the in vivo survival and virulence of diverse human and plant pathogens (Figure 1). With the technological advancement and genome editing approaches such as CRISPR-Cas9, in the future, additional novel roles of GlcNAc as a signaling molecule and GlcNAc engineering will continue to emerge in a diverse range of cell types. In the long term, enzymes and genes of the GlcNAc metabolic pathway could be used for the drug targets for controlling various human and plant diseases. 

An assessment of the infectious host-pathogen interaction model has shown the virulence and defense mechanisms related to GlcNAc engineering. Although, there is still a scarcity in model pathosystems to examine GlcNAc function or GlcNAc perception by the host during pathological interactions. Hence, imminent improvement in this field will illustrate the critical role of GlcNAc signaling/perception during pathological interactions and also extend the co-adaptation of the pathogen, subsequently allowing pathogen invasion and host colonization. Indeed, such discernments will not only assist our understanding of advantageous versus disadvantageous communications but could also encourage resistance breeding in the future. Therefore, when new carbohydrate-based prospective DAMPs/MAMPs become accessible with the understanding of GlcNAc signaling/perception, we may be able to raise crop varieties harboring specific plant pattern-recognition receptors and also design agricultural approaches that would augment crop disease resistance and modulate crop immunity. 

## Figures and Tables

**Table 1:** Microorganisms and associated host cell surface protein and pathogen infection at mucosal membrane. Mucous membrane is rich in glycosylated proteins and several pathogens, such as *Candida albicans*, *Escherichia coli*, *Salmonella*, *Vibrio cholerae*, etc., exploit glycoproteins at the mucosal membrane.

Microbes	Cell Surface Protein	Organ/Tissue	Disease	References
*Helicobacter pylori*	Mucin 5AC (MUC5AC)	Gastric mucosa	Peptic ulcers	[36]
*Vibrio cholerae*	N-acetylneuraminic acid (Neu5Ac) and N-acetylglucosamine (GlcNAc)	Small intestine	Cholera	[37]
*Salmonella enterica*	MUC1	Intestinal epithelial cells	Typhoid	[38]
*Leishmania* species	Neuraminidase 1 (NEU 1)	Skin, spleen, and liver	Leishmaniasis	[39]
*Toxoplasma gondii*		Spleen, lung, etc.	Toxoplasmosis	[40,41]
Enterotoxigenic *Escherichia coli*	MUC2	Large intestine	Noninflammatory Diarrheas	[42]
*Candida albicans*	Msb2	Mouth, throat, gut, and vagina	Candidiasis	[43,44]
*Akkermansia muciniphila*	Gastrointestinal mucin	Gastrointestinal tract	-	[45,46]
*Clostridioides difficile*	O-glycan mucin	Gastrointestinal tract	Diarrhea	[47]
*Staphylococcus aureus*	Nasal mucin	Brain, heart, and lung	Pneumonia and Meningitis	[48,49]

## Data Availability

Not applicable.

## Abbreviations

Avr9	Avirulence gene
Camp	Cyclic adenosine monophosphate
CBLs	Chitin-binding lectins
CERK1	Chitin elicitor receptor kinase 1
CRISPR	Clustered regularly interspaced short palindromic repeats
DAC	N-acetyl glucosamine-6-phosphate deacetylase
DAMPs	Damage-associated molecular patterns
ECM	Extracellular Matrix
GI	Gastrointestinal
GlcNAc-1-P	N-acetylglucosamine-1-phosphate
GlcNAc-6-P	N-acetylglucosamine-6-phosphate
GlcNAc	N-acetylglucosamine
HXK	Hexokinase
LYK	LysM domain receptor-like kinase
lyk3	LysM domain receptor-like kinase 3
LysM	Lysin motif domain
MAMPs	Microbe-associated molecular patterns
NAG	glucosamine-6-phosphate deaminase
NFP	Nod factor perception
PRR	Pattern recognition receptor
PTS	Phosphotransferase system
PV	Parasitophorous vacuole
ROS	Reactive oxygen species
UDP-GlcNAc	Uridine diphosphate N-acetylglucosamine
